# Supplementary material for: The Unrealised Potential for Predicting Pregnancy Complications in Women with Gestational Diabetes: A Systematic Review and Critical Appraisal
Source: Int J Environ Res Public Health. 2020 Apr 27;17(9):3048. doi: 10.3390/ijerph17093048 (PMC7246772; doi:10.3390/ijerph17093048)
Supplement: Supplementary file 1 [file ijerph-17-03048-s001.pdf]

Supplementary Material

Table S1. Search strategy for MEDLINE.

Concept	Step	Search Strategy
**Study type = prediction modelling studies**		
Study type = prediction modelling studies (Ingui filter for prediction models[1])	1	Validat$.mp. or Predict$.ti. or Rule$.mp.
	2	(Predict$ and (Outcome$ or Risk$ or Model$)).mp.
	3	((History or Variable$ or Criteria or Scor$ or Characteristic$ or Finding$ or Factor$) and (Predict$ or Model$ or Decision$ or Identif$ or Prognos$)).mp.
	4	Decision$.mp. and ((Model$ or Clinical$).mp. or Logistic Models/)
	5	(Prognostic and (History or Variable$ or Criteria or Scor$ or Characteristic$ or Finding$ or Factor$ or Model$)).mp.
	6	or/1-5
Study type = prediction modelling studies (Addition to Ingui filter proposed by Geersing to improve sensitivity[2])	7	Stratification.mp. or "ROC Curve"/ or Discrimination.mp. or Discriminate.mp. or c-statistic.mp. or "c statistic".mp. or "Area under the curve".mp. or AUC.mp. or Calibration.mp. or Indices.mp. or Algorithm.mp. or Multivariable.mp. [mp=title, abstract, original title, name of substance word, subject heading word, floating sub-heading word, keyword heading word, protocol supplementary concept word, rare disease supplementary concept word, unique identifier, synonyms]
Combination of study type concepts	8	6 or 7
**Population = women with gestational diabetes**		
	9	exp diabetes, gestational/
	10	(gestation* adj4 diabet*).ti,ab.
	11	gdm.ti,ab.
	12	(glucose adj4 (pregnan* or gestation* or prenatal* or antenatal* or pre-natal* or ante-natal* or maternal*)).ti,ab.
	13	or/9-12
**Outcomes = pregnancy complications**		
Hypertensive disorders of pregnancy	14	exp Hypertension, Pregnancy-Induced/
	15	((hypertens* adj3 pregnan*) or gestational hypertens* or pre-eclamp* or preeclamp* or eclamp* or HELLP syndrome).ti,ab.
	16	14 or 15
Perinatal mortality	17	exp Perinatal Mortality/ or Fetal Mortality/ or Perinatal Death/ or exp Fetal Death/
	18	(((perinatal or neonatal or f?etal or intrauterine or intra-uterine) adj (mortalit* or death* or demise or loss)) or stillbirth*).ti,ab.
	19	17 or 18
Large-for-gestational age (LGA)	20	exp Birth Weight/
	21	((Large* adj3 gestation*) or LGA or large birth*).ti,ab.
	22	20 or 21

Perinatal morbidity	23	exp Birth Injuries/ or exp $Dystocia/ or exp Brachial Plexus Neuropathies/ or exp Obstetric Labor Complications/
	24	(birth injur* or ((birth or labo?r or obstetric* or perinatal or delivery) adj4 (trauma* or injur* or complication*1)) or (shoulder adj4 dystocia*) or (fracture*1 adj4 (clavic* or humerus or shoulder*1 or arm*1)) or ((neonat* or infant* or erb* or obstetric or brachial) adj5 (palsy or neuropath*)) or nerve palsy or plexus injury).ti,ab.
	25	23 or 24
Caesarean delivery	26	exp Cesarean Section/
	27	(c?esar* or (delivery adj abdominal) or c-section).ti,ab.
	28	26 or 27
Maternal mortality	29	Maternal Mortality/ or Maternal Death/
	30	((maternal or mother* or woman or women or pregnan* or gestation*) adj2 (mortalit* or death* or dies or died)).ti,ab.
	31	29 or 30
Induction of labour	32	exp Labor, Induced/
	33	(induc* adj3 (labo?r or deliver*)).ti,ab.
	34	32 or 33
Placental abruption	35	Abruptio Placentae/
	36	(placenta* adj2 abrupt*).ti,ab.
	37	35 or 36
Perineal trauma	38	Episiotomy/
	39	((perine* adj2 (trauma or injur* or tear*)) or ((birth or sphincter*) adj (injury or trauma)) or (third-degree adj tear*) or (fourth-degree adj tear*) or episiotom*).ti,ab.
	40	38 or 39
Postpartum haemorrhage	41	Postpartum Hemorrhage/
	42	((postpartum or post-partum) adj1 h?emorrhage).ti,ab.
	43	41 or 42
Neonatal hypoglycaemia	44	hypoglycemia/ and (infan* or neonat* or newborn* or baby or babies).ti,ab.
	45	(((infan* or neonat* or newborn* or baby or babies) and hypoglyc?em*) or (((infan* or neonat* or newborn* or baby or babies) and low) adj2 (glucose or sugar))).ti,ab.
	46	44 or 45
Fetal macrosomia	47	exp Fetal Macrosomia/
	48	macrosomi*.ti,ab.
	49	47 or 48
Small-for-gestational age	50	Infant, Small for Gestational Age/
	51	((small* adj3 gestation*) or SGA).ti,ab.
	52	50 or 51
Low birth weight	53	exp Infant, Low Birth Weight/
	54	(low-birth-weight or low birthweight*).ti,ab.
	55	53 or 54

Intrauterine growth restriction (IUGR)	56	exp Fetal Growth Retardation/
	57	(fetal growth restrict* or fetal growth retard* or intra-uterine growth retard* or intra-uterine growth restrict* or intrauterine growth retard* or intrauterine growth restrict* or IUGR).ti,ab.
	58	56 or 57
Preterm delivery or premature birth	59	exp Obstetric Labor, Premature/ or exp infant, premature/
	60	(((pre-term* or preterm* or premature) adj birth*) or premat*).ti,ab.
	61	59 or 60
Admission to neonatal intensive care unit	62	Intensive Care, Neonatal/ or Intensive Care Units, Neonatal/
	63	(((neonatal or newborn*) adj1 (intensive or unit* or ICU*)) or ((neonatal or special) adj2 nursery) or NICU).ti,ab.
	64	62 or 63
Neonatal respiratory distress syndrome	65	Respiratory Distress Syndrome, Newborn/
	66	((infan* or neonat* or newborn* or baby or babies) and (respiratory distress or dyspn?ea or cyanosis or RDS)).ti,ab.
	67	65 or 66
Neonatal jaundice	68	exp Hyperbilirubinemia, Neonatal/
	69	((infan* or neonat* or newborn* or baby or babies) adj5 (hyperbilirubin* or jaundice)).ti,ab.
	70	68 or 69
Neonatal hypocalcaemia	71	Hypocalcemia/ and (infan* or neonat* or newborn* or baby or babies).ti,ab.
	72	((infan* or neonat* or newborn* or baby or babies) and (hypocalc?emi* or (low adj2 calcium))).ti,ab.
	73	71 or 72
Neonatal adiposity	74	Pediatric obesity/ and (infan* or neonat* or newborn* or baby or babies).ti,ab.
	75	(((infan* or neonat* or newborn* or baby or babies) adj5 (adipos* or skin-fold or skinfold or weight or body mass index or fat or abdominal circumference or obesity or anthropometr*)) or ponderal index).ti,ab.
	76	74 or 75
Postpartum infection	77	Puerperal Infection/
	78	((postpartum* or post-partum* or puerper*) and (infect* or sepsi*)).ti,ab.
	79	77 or 78
Apgar score < seven at 5 minutes	80	Apgar Score/
	81	apgar*.ti,ab.
	82	80 or 81
Neonatal polycythaemia	83	Polycythemia/ and (infan* or neonat* or newborn* or baby or babies).ti,ab.
	84	((infan* or neonat* or newborn* or baby or babies) adj3 polycyth?emia).ti,ab.
	85	83 or 84
Instrumental delivery	86	exp Extraction, Obstetrical/ or exp Delivery Obstetric/ or exp Vacuum Extraction, Obstetrical/

	87	((obstetric* or instrument* or forcep* or vacuum*) adj2 (deliver* or extraction*)).ti,ab.
	88	86 or 87
'Pregnancy complications' as a general concept	89	exp Pregnancy Outcome/ or exp Pregnancy Complications/
	90	((pregnan* or perinatal or obstetric* or labo?r or birth or neonatal or maternal) adj3 (outcome* or complication* or adverse)).ti,ab.
	91	89 or 90
**Combination of all pregnancy complication concepts**	92	16 or 19 or 22 or 25 or 28 or 31 or 34 or 37 or 40 or 43 or 46 or 49 or 52 or 55 or 58 or 61 or 64 or 67 or 70 or 73 or 76 or 79 or 82 or 85 or 88 or 91
**Combination of concepts**	93	8 and 13 and 92
**Human filter**	94	exp animals/ not humans/

Table S2. Model development characteristics of models for pregnancy complications in women with gestational diabetes.

Model	Presence of Missing Data and Handling	Selection of Predictors for Inclusion in Modelling	Modelling Method	Selection of Predictors During Multivariable Modelling	Shrinkage of Predictor Weights or Regression Coefficients
A risk engine relating maternal glycaemia and body mass index to pregnancy outcomes (McIntyre *et al.*)[3]	Missing data for some independent variables reported but not quantified; participants excluded if missing data for these predictors.	Based on exploratory analyses	LR	Full-model approach (includes all candidate predictors)	NR
Screening tool for predicting adverse outcomes of GDM (Park *et al.*)[4]	NR	Pre-selection based on unadjusted association with the outcome	LR	NR	NR
A risk score based on clinical characteristics of GDM women for the development of preeclampsia (Phaloprakarn and Tangjitgamol)[5]	NR	Pre-selection based on unadjusted association with the outcome	LR	NR	NR
Subgroups at different risks of developing the composite adverse neonatal outcome (Pintaudi *et al.*)[6]	NR	NR	RECPAM	Included in RECPAM method	NR
The fetal overgrowth index (Tomlinson *et al.*)[7]	Represents < 1% of data for predictors included in the final model; handling not reported.	Pre-selection based on unadjusted association with the outcome	LR	Forward stepwise method	NR

Abbreviations: LR, logistic regression; NR, not reported; GDM, gestational diabetes; RECPAM, RECursive Partitioning and AMalgamation.

Table S3. The risk of bias and concern regarding the applicability of the models developed in the five prediction modelling studies using the Prediction model Risk of Bias Assessment Tool.

Model	Risk of Bias				Concern Regarding Applicability			Overall	
	Participants	Predictors	Outcome	Analysis	Participants	Predictors	Outcome	Risk of Bias	Concern Regarding Applicability
A risk engine relating maternal glycaemia and body mass index to pregnancy outcomes (McIntyre *et al.*)[3]	High	Low	Low	High	High	Low	Low	High	High
Screening tool for predicting adverse outcomes of GDM (Park *et al.*)[4]	High	Low	Low	High	High	Low	Low	High	High
A risk score based on clinical characteristics of GDM women for the development of preeclampsia (Phaloprakarn and Tangjitgamol)[5]	Low	Low	Unclear	High	Low	Low	Unclear	High	Unclear
Subgroups at different risks of developing the composite adverse neonatal outcome (Pintaudi *et al.*)[6]	Low	Low	Low	High	Low	Low	Low	High	Low
The fetal overgrowth index (Tomlinson *et al.*)[7]	Low	Low	Low	High	Low	Low	Low	High	Low

References

1. Ingui, B.J.; Rogers, M.A.M. Searching for Clinical Prediction Rules in Medline. *J. Am. Med Informatics Assoc.* **2001**, *8*, 391–397, doi:10.1136/jamia.2001.0080391.
2. Geersing, G.J.; Bouwmeester, W.; Zuithoff, P.; Spijker, R.; Leeflang, M.; Moons, K.G. Search Filters for Finding Prognostic and Diagnostic Prediction Studies in Medline to Enhance Systematic Reviews. *PLOS ONE* **2012**, *7*, e32844, doi:10.1371/journal.pone.0032844.
3. McIntyre, H.D.; Gibbons, K.; Lowe, J.; Oats, J.J. Development of a risk engine relating maternal glycemia and body mass index to pregnancy outcomes. *Diabetes Res. Clin. Pr.* **2018**, *139*, 331–338, doi:10.1016/j.diabres.2018.02.036.
4. Park, J.S.; Kim, D.W.; Kwon, J.Y.; Park, Y.W.; Kim, Y.-H.; Cho, H.Y. Development of a Screening Tool for Predicting Adverse Outcomes of Gestational Diabetes Mellitus. *Med.* **2016**, *95*, e2204, doi:10.1097/MD.0000000000002204.
5. Phaloprakarn, C.; Tangjitgamol, S. Risk assessment for preeclampsia in women with gestational diabetes mellitus. *J. Périnat. Med.* **2009**, *37*, 617–621, doi:10.1515/jpm.2009.108.
6. Pintaudi, B.; Fresa, R.; Dalfrà, M.; Dodesini, A.R.; Vitacolonna, E.; Tumminia, A.; Sciacca, L.; Lencioni, C.; Marcone, T.; Lucisano, G., et al. The risk stratification of adverse neonatal outcomes in women with gestational diabetes (STRONG) study. *Acta Diabetol.* **2018**, *55*, 1261–1273, doi:10.1007/s00592-018-1208-x.
7. Tomlinson, T.M.; Mostello, D.J.; Lim, K.-H.; Pritchard, J.S.; Gross, G. Fetal overgrowth in pregnancies complicated by diabetes: development of a clinical prediction index. *Arch. Gynecol. Obstet.* **2018**, *298*, 67–74, doi:10.1007/s00404-018-4758-9.

© 2020 by the authors. Licensee MDPI, Basel, Switzerland. This article is an open access article distributed under the terms and conditions of the Creative Commons Attribution (CC BY) license (http://creativecommons.org/licenses/by/4.0/).
